# Supplementary material for: WordCommentsAnalyzer: A windows software tool for qualitative research
Source: F1000Res. 2018 Sep 4;7:536. Originally published 2018 May 3. [Version 2] doi: 10.12688/f1000research.14819.2 (PMC6137411; doi:10.12688/f1000research.14819.2)
Supplement: Supplementary file 3 [file f1000research-7-17614-s0002.tgz › b2a79496-4ada-47c4-9682-a99ce867836d.zip!/Eight Iranian Health Journals/Journal of Health System Research _26.docx]

© Journal of Health System Research . This work is licensed under CC BY-NC 4.0 (https://creativecommons.org/licenses/by-nc/4.0/).

Volume & Issue: دوره 9، شماره 6؛ 1392

Title: ادراک افراد مبتلا به پرفشاری خون از بیماری و درمان آن: یک مطالعه مروری

http://hsr.mui.ac.ir/index.php/jhsr/article/view/864

مقدمه: پرفشاری خون یک مشکل بزرگ بهداشتی در کشورهای توسعه یافته و در حال توسعه است و همه‌گيري رو به افزایش این بیماری در جهان هشداري جدي براي توجه بيشتر به اين بيماري می‌باشد که به طور عمده خاموش است. این مطالعه با هدف، بررسی ادراک بیماران مبتلا به پرفشاری خون از بیماری و درمان آن در مقالات منتشر شده انجام شد. روش‌ها: مطالعه حاضر از نوع مروری سیستماتیک بود و جستجوی مقالات کیفی در پايگاه‌های اطلاعاتی ايرانی و بين‌المللی Iran Medex ، SID، PubMed، ISI web of knowledge، Embase،  Medlineو Google Scholar با کليد واژه‌های «پرفشاری خون و پیگیری درمان» و در محدوده زمانی سال‌های 1980 تا 2012 ميلادی انجام گرفت. معیار ورود مقالات به مطالعه شامل کیفی بودن مطالعه، بررسی دیدگاه‌ها، ادراک و تجارب بیماران مبتلا به پرفشاری خون و انتشار مقاله به زبان فارسی و انگلیسی بود. مطالعاتی که بیماران مبتلا به بیماری‌های مزمن دیگر مانند دیابت و بیماری‌های قلبی- عروقی را در برمی‌گرفت و مطالعات مربوط به پرفشاری خون دوران بارداری از بررسی خارج شدند. یافته‌ها: از تعداد 57 مقاله یافت شده، تعداد 38 مقاله در این مطالعه مورد بررسی قرار گرفتند و یافته‌های حاصل از این مقالات در قالب دو درون‌مایه: الف. پرفشاری خون از دیدگاه بیماران و ب. پیگیری درمان، دیدگاه‌ها و دلایل به دست آمدند. استرس به عنوان یک عامل و پیامد ناشی از بیماری مورد توجه بود. بیماران ادراک منفی نسبت به درمان داشته و آن را نامطلوب می‌دانستند و در برخی موارد وجود یا عدم وجود علایم بالینی عاملی برای قطع یا کاهش مصرف دارو محسوب می‌شد. عدم مصرف و یا کاهش آن دلایل غیر عمدی نیز داشت که شایع‌ترین آن‌ها، فراموشی مصرف دارو، نداشتن وقت و اشتغال بیش از حد، هزینه زیاد دارو و ویزیت پزشکی و تغذیه درمانی و نداشتن بیمه بود. نتيجه‌گيری: در برخی موارد بیمار به طور عمد و با آگاهی کامل تصمیم به قطع و یا کاهش دارو می‌گیرد و این مسأله می‌تواند توجیهی برای اثربخشی کوتاه مدت و یا بی‌اثر بودن مداخلات درمانی و آموزشی باشد. بنابراین توجه به نگرانی‌ها و ادراک بیماران در مداخلات برای موفقیت در درمان و کنترل بیماری ضروری است.

Title: بررسی نقش تحریکات حسی در تغییر سطح هوشیاری بیماران در وضعیت کما (مقاله مروری)

http://hsr.mui.ac.ir/index.php/jhsr/article/view/1249

محرومیت حسی که به دنبال کاهش سطح هوشیاری در بیماران در وضعیت کما ایجاد می‌شود؛ ضمن اختلال در روند بهبودی ضایعه عصبی، باعث بستری طولانی مدت بیماران شده و هزینه‌های زیادی را به خانواده و جامعه تحمیل نموده و کیفیت زندگی بیمار و خانواده را تحت تأثیر قرار می‌دهد. مداخلات تحریک حسی یکی از روش‌های توان‌بخشی بیماران در وضعیت کما است که با هدف افزایش سطح هوشیاری و بازگشت سریع‌تر بیماران به جامعه انجام می‌شود. هدف از این مطالعه، تعیین نقش تحریکات حسی در تغییر سطح هوشیاری بیماران بیهوش از طریق مرور مطالعات انجام شده در این راستا بود. مطالعه حاضر از نوع مروری بود که برای انجام آن مقالات بین سال‌های 2012-2000 به زبان‌های فارسی و انگلیسی در پایگاه‌های علمی جستجو و بررسی شدند و 49 مطالعه با کلید واژه‌های مربوط حاصل گردید. از این تعداد، 14 مطالعه دارای معیارهای ورود بودند. محققین بر اساس روش کار خود از یک یا چند تحریک حسی استفاده نمودند. از تحریک حسی شنوایی در 100 درصد مطالعات استفاده شده بود. 93 درصد مطالعات تأثیر تحریکات حسی بر افزایش سطح هوشیاری بیماران در وضعیت کما را تأیید نمودند و 7 درصد تحریکات حسی را بر افزایش سطح هوشیاری بیماران بی‌تأثیر دانسته بودند. ارایه تحریکات حسی به بیماران بیهوش باعث افزایش سطح هوشیاری آن‌ها می‌شود. پرستاران به عنوان افرادی که در تماس مداوم با بیماران هستند باید با کمک گرفتن از اعضای خانواده محیطی غنی از تحریکات حسی را برای بیماران فراهم نمایند.

Title: بررسی ارتباط افسردگی پس از زایمان با تغذیه انحصاری با شیرمادر

http://hsr.mui.ac.ir/index.php/jhsr/article/view/844

مقدمه: در سال‌های اخیر، شاخص‌های مربوط به تغذیه انحصاری با شیر مادر در ایران افت داشته است. از آن جا که شیردهی انحصاری با شیر مادر به فاکتورهای متعددی بستگی دارد، شناخت هر یک از این عوامل تأثیرگذار در اتخاذ استراتژی‌های پیشگیرانه از قطع زود هنگام تغذیه انحصاری با شیر مادر، کمک کننده خواهد بود. این مطالعه، با هدف تعیین ارتباط بین افسردگی پس از زایمان با تغذیه انحصاری با شیر مادر صورت گرفته است.  روش‌ها: این مطالعه مقطعی از نوع مورد- شاهد، در طی شش ماه اول سال 1391 در شهر قم صورت گرفت. مادران به 3 گروه 100 نفره بر حسب وضعیت تغذیه نوزاد خود تقسیم شدند. در این مطالعه، جهت خنثی کردن متغیرهای دخیل در افسردگی، از روش‌های محدودسازی و همسان‌سازی استفاده شد. در این مطالعه جهت گردآوری داده‌ها، علاوه بر پرسش‌نامه مشخصات دموگرافیک، از پرسش‌نامه استاندارد Beck نیز استفاده شد. داده‌ها در سطح معنی‌داری کمتر از 05/0 تحلیل گردید.  یافته‌ها: میانگین نمره افسردگی در گروه تغذیه انحصاری با شیر مادر 81/12، در گروه تغذیه تکمیلی همزمان با شیر مادر 59/13 و در گروه قطع کامل شیردهی 06/15 نمره بود که اختلاف معنی‌داری بین گروه‌ها یافت نشد. افسردگی خفیف در گروه تغذیه انحصاری با شیر مادر 5 درصد، در گروه تغذیه تکمیلی همزمان با شیر مادر 8 درصد و در گروه قطع کامل شیردهی 10 درصد بود. همچنین افسردگی شدید فقط در گروه قطع کامل شیردهی به میزان 5 درصد مشاهده شد. همچنین بین افسردگی با کفایت شیر و حمایت همسر، ارتباط معکوس معنی‌دار مشاهده گردید. نتیجه‌گیری: اگر چه میزان افسردگی در گروه انحصاری تغذیه با شیر مادر کمتر از سایر گروه‌ها بود، اما آزمون آنالیز واریانس، اختلاف معنی‌داری را در این خصوص نشان نداد. همچنین درک مادران از کفایت شیر و نیز حمایت همسر، موجب کاهش میزان افسردگی می‌شد.

Title: بررسی میزان آگاهی و باور دانش‌آموزان پسر مقطع پیش‌دانشگاهی شهر اصفهان در خصوص استعمال سیگار و مضرات آن و شیوع مصرف سیگار در بین آنان

http://hsr.mui.ac.ir/index.php/jhsr/article/view/859

مقدمه: یکی از رفتارهای غیر بهداشتی که سلامتی افراد جامعه را به خطر می‌اندازد و هزینه‌های قابل توجهی را بر جوامع تحمیل می‌کند، مصرف سیگار است. نظر به این که میزان استعمال سيگار در ميان جوانان و نوجوانان در حال افزايش و سن شروع آن رو به كاهش مي‌رود، مطالعه حاضر با هدف تعیین شیوع مصرف سیگار و میزان آگاهی و نگرش دانش‌آموزان پسر مقطع پیش دانشگاهی شهر اصفهان در خصوص استعمال سیگار و مضرات آن انجام پذیرفت. روش‌ها: این مطالعه توصیفی- تحلیلی و به شیوه مقطعی بر روی 382 دانش‌آموز پسر مقطع پیش دانشگاهی که با روش نمونه‌گیری تصادفی سیستماتیک انتخاب شده بودند، انجام شد. ابزار گردآوری داده‌ها، پرسش‌نامه محقق ساخته‌ای مشتمل بر 3 بخش (مشخصات فردی، آگاهی و نگرش) بود. داده‌های گردآوری شده، توسط نرم‌افزار SPSS نسخه 18، آمار توصیفی و آزمون Chi-square مورد تجزیه و تحلیل قرار گرفت. یافته‌ها: میانگین سنی دانش‌آموزان 62/0 ± 72/17 سال بود. 33 درصد از افراد، تجربه حداقل یک بار سیگار کشیدن را داشتند. 2/7 درصد واحدهای مورد پژوهش، سیگاری بودند. 6/41 درصد از افراد مورد پژوهش از نگرش خوبی برخوردار بودند، در حالی که تنها 3/18 درصد از افراد، از سطح آگاهی خوبی برخوردار بودند. در افراد با سطح آگاهی بالاتر، تجربه استعمال سیگار کمتر بود (017/0 = P). همچنین دانش‌آموزانی که تجربه مصرف سیگار را گزارش نمودند، نگرش ضعیف‌تری در خصوص مضرات سیگار کشیدن داشتند (001/0 = P). نتیجه‌گیری: پایین بودن سطح آگاهی و نگرش دانش‌آموزان، پایین بودن سن شروع مصرف سیگار و همچنین بالا بودن نسبی تجربه مصرف سیگار در این افراد هشدار دهنده می‌باشد. بر این اساس، اقدامات پيشگيري در دوره نوجواني و قبل از آن، ضروری به نظر می‌رسد.

Title: چگونه بروز خطاهای دارویی و عدم گزارش آن توسط پرستاران را در بخش مرقبت‌های ویژه قلبی کاهش دهیم؟ یک مطالعه اقـدام پژوهی

http://hsr.mui.ac.ir/index.php/jhsr/article/view/884

مقدمه: خطاهای ‌دارویی یکی ازشايع‌ترين حوادث‌ موجود در حرفه ‌پرستاري می‌باشد که برای مددجویان بالقوه‌ خطرناک است. هدف از ‌این مطالعه طراحی ‌و اجرای راهکارهای‌ کاهش بروز خطاهای دارویی و عدم گزارش آن توسط پرستاران در بخش مراقبت‌های ویژه ‌قلب در یکی از مراکز بیمارستانی وابسته به دانشگاه علوم پزشکی اصفهان بود.  روش‌ها: ‌این پژوهش یک ‌مطالعه اقدام ‌پژوهشی‌ بود که ‌در سال 1389 انجام‌ شد. مشارکت‌کنندگان تمامی پرسنل بخش‌ ویژه قلب یکی از بیمارستان‌های وابسته به دانشگاه ‌علوم ‌پزشکی ‌اصفهان بودند که به ‌روش‌ سرشماری وارد مطالعه‌‌ شدند. جهت‌ جمع‌آوری اطلاعات از پرسش‌نامه ‌خودساخته ‌چهاربخشی استفاده ‌شد. روایی و پایایی آن به ترتیب توسط روش ‌اعتبار محتوی و روش ‌آزمون مجدد تعیین‌ شد. سپس پژوهشگران به بررسی راهکارهای موجود و انتخاب ‌بهترین ‌راهکارها بر اساس جدول Matrix   و Thomas پرداختند. پس ازعملیاتی ‌نمودن راهکارهای منتخب در بخش مورد نظر، نمونه‌های ‌مورد پژهش ‌سـه ‌ماه ‌بعد با همان‌ پرسش‌نامه، مجدد مورد ارزیابی ‌قرار گرفتند. داده‌ها با استفاده ‌از نرم‌افزار SPSS نسخه 16 و به کارگیری ‌آمار توصیفی، متد آماری Paired t و ضریب همبستگی Pearson مورد تجزیه‌ و تحلیل قرار گرفت. یافته‌ها: گویه‌های «عدم انجام احتیاط‌های دارویی» با 30 درصد، «دادن دوز اشتباه دارو» با 25 درصد و «سرعت انفوزیون اشتباه» با 25/21 درصد بیشترین خطاهای دارویی گزارش شده توسط پرسنل بودند. از نظرعلل بروز خطاهای ‌دارویی، همه ‌پاسخ‌دهندگان کم بودن نسبت پرستار به بیمار، تنوع ‌زیاد داروها، تعداد زیاد بیماران ‌بدحال و بار کاری بالا را عامل بروز خطاهای‌ دارویی ذکر کردند. عدم وجود سیستم ثبت خطاها (84 درصد)، عدم‌ آگاهی از تعریف‌ خطای دارویی (81 درصد)، ترس‌ از مسایل ‌قضایی (80 درصد)، وقت‌گیر بودن (73 درصد) و عدم حمایت کافی سیستم از پرسنل (68 درصد) از شایع‌ترین علل عدم‌ گزارش‌ خطاهای‌ دارویی به‌ وقوع پیوسته بود. پس از انتخاب و عملیاتی ‌شدن‌ راهکارهای منتخب در طی 3 ماه، ‌میانگین بروز خطای ‌دارویی برای هر پرسنل از 09/2 ± 5/12 به 02/1 ± 09/5 رسید که میزان‌ کاهش‌ وقوع خطاهای ‌دارویی از نظر آماری معنی‌دار بود (02/0 =P ). همچنین نتایج آزمون Paired t نشان داد که بین میانگین نمره گزارش اشتباهات دارویی قبل و بعد از اجرایی شدن راهکارها از نظر آماری تفاوت معنی‌داری داشت (04/0 =P )، به ‌طوری‌ که میانگین گزارش خطا از 41/0 ± 1/1 برای هر پرسنل به 51/0 ± 1/3 افزایش یافت. نتیجه‌گیری: بر اساس یافته‌های پژوهش جهت ارتقای کمی‌ و کیفی دانش ‌پرستاران نسبت به مقوله خطاهای ‌دارویی، راهکارهایی همچون ترویج فرهنگ استفاده ازکتابچه‌های اطلاعات ‌دارویی در بخش، دایر نمودن‌ کلاس ‌بازآموزی پیرامون اطلاعات‌ دارویی برای پرسنل ‌بالینی به شکل ‌منظم، سهولت‌ دسترسی به سایت‌های‌ کامپیوتر و شبکه ‌اینترنتی در مراکز درمانی جهت ‌ارتقا و به‌‌روز شدن اطلاعات ‌دارویی پرستاران و ارزیابی مستمر دانش و اطلاعات ‌دارویی پرسنل بخش مورد عنایت و توجه بیشتری قرار گیرد.

Title: بررسی ارتباط بین نتایج حاصل از قدم‌سنج با پرسش‌نامه بین‌المللی فعالیت بدنی IPAQ

http://hsr.mui.ac.ir/index.php/jhsr/article/view/897

مقدمه: فعالیت بدنی منظم در کاهش خطر ابتلا به بیماری‌ها و مرگ و میر تأثیرگذار است. مطالعه‌ها نشان دادند که رسیدن به 10000 قدم در روز در ارتقای فعالیت بدنی و رسیدن به پیامدهای سلامتی مؤثر است. هدف از این مطالعه مقایسه فعالیت بدنی گزارش شده از طریق پرسش‌نامه با فعالیت بدنی گزارش شده از قدم‌سنج و مقایسه یافته‌های حاصل از قدم‌سنج با راهنمای رسیدن به 30 دقیقه فعالیت بدنی متوسط یا شدید در هفته بود. روش‌ها: این مطالعه مقطعی بود. مطالعه روی 200 نفر از کارمندان زن دانشگاه تبریز صورت گرفت که به روش تصادفی آسان در دسترس انتخاب شدند. ابزار جمع‌آوری داده‌ها شامل پرسش‌نامه بین‌المللی فعالیت بدنی جهت ارزیابی فعالیت بدنی ذهنی و قدم‌سنج برای اندازه‌گیری فعالیت بدنی عینی بود. يافته‌ها: بر اساس نتایج قدم‌سنج تقریباً نیمی از شرکت‌کنندگان کم تحرک بودند، در حالی که نتایج International Physical Activity Questionnaire (IPAQ) نشان داد که 6/86 درصد از شرکت‌کنندگان فعال بودند و به راهنمای 30 دقیقه فعالیت رسیدند. بنابراین تطابقی بین نتایج این دو ابزار وجود نداشت. همچنین بین نتایج حاصل از ابزار عینی و ذهنی فعالیت همبستگی معنی‌داری دیده نشد. نتيجه‌گيری: این مطالعه احتمالاً اولین مطالعه‌ای است که در ایران فعالیت بدنی زنان را به شکل عینی با استفاده از قدم‌سنج ارزیابی و با پرسش‌نامه مقایسه نموده است. نتایج نشان داد که خود گزارش‌دهی فعالیت بدنی بر اساس پرسش‌نامه بالا است. نتایج بین ابزار عینی و ذهنی فعالیت بدنی با هم تطابقی نداشتند. می‌توان گفت که پژوهشگران در بررسی نتایج حاصل از IPAQ باید میزان گزارش بالای فعالیت را در نظر بگیرند.

Title: ارزیابی بار ذهنی کار در پرستاران بخش مراقبت ویژه با استفاده از شاخص بار ذهنی (NASA)

http://hsr.mui.ac.ir/index.php/jhsr/article/view/930

مقدمه: یکی از عوامل تأثیرگذار بر رفتار، عملکرد و کارایی پرستاران در محیط کار، بار ذهنی کار می‌باشد. از این رو توجه به آن از اهمیت بسزایی برخوردار است. هدف مطالعه حاضر ارزیابی بار ذهنی کار و عوامل مؤثر بر آن در پرستاران بخش مراقبت‌های ویژه بود. روش‌ها: این مطالعه به صورت توصیفی- تحلیلی و از نوع مقطعی بود که روی 163 نفر از پرستاران بخش مراقبت ویژه شاغل در بیمارستان‌های شهر اصفهان انجام گرفت. جهت جمع‌آوری داده‌ها از پرسش‌نامه تخصصی بار ذهنی National aeronautics and space administration یا NASA (NASA-TLX) استفاده شد. اطلاعات به دست آمده از پرسش‌نامه وارد نرم‌افزار بار ذهنی NASA شد و نمره نهایی بار ذهنی تعیین گردید. داده‌ها با استفاده از نرم‌افزار آماری SPSS نسخه 18 و با استفاده از آزمون‌های آماری همبستگی Pearson و آنالیز One Way ANOVA تجزیه و تحلیل شد. یافته‌ها: آزمون همبستگی Spearman نشان داد که بین متغیرهای زمینه‌ای با بار ذهنی رابطه معنی‌داری وجود نداشت، اما بین تعداد بیماران تحت نظر فرد در هر شیفت کاری و نمره بار ذهنی پرستاران ارتباط معنی‌داری به صورت مستقیم یافت شد (05/0 > P). همچنین شاخص NASA نشان داد که کمترین نمره مقیاس‌های شش‌گانه بار ذهنی در پرستاران مربوط به نیاز زمانی (56/63) و بیشترین نمره مربوط به نیاز ذهنی (50/83) بود. نتیجه‌گیری: نتایج حاکی از بالا بودن بار ذهنی درک‌شده ناشی از ارایه خدمات در پرستاران مورد مطالعه بود. از این رو به کارگیری استراتژی‌های مداخله‌ای برای کاهش بار ذهنی در این گروه ضروری به نظر می‌رسد.

Title: بررسی عادات تغذیه‌ای بیماران مبتلا به دیابت نوع 2 مراجعه کننده به بیمارستان‌های تابعه دانشگاه علوم پزشکی تهران

http://hsr.mui.ac.ir/index.php/jhsr/article/view/1212

مقدمه: اتخاذ رفتارهای مطلوب تغذیه‌ای یک بخش اصلی در بیماران مبتلا به دیابت نوع 2 است که می‌تواند به پیشگیری و کاهش عوارض ناشی از بیماری بیانجامد. شواهد نشان می‌دهد که بهبود عادات و رفتارهای تغذیه‌ای می‌تواند به کنترل دیابت کمک نماید. این مطالعه با هدف، تعیین عادات تغذیه‌ای دیابت در بیماران مبتلا به دیابت نوع 2 مراجعه کننده به بیمارستان‌های تابعه دانشگاه علوم پزشکی تهران در سال 1391 طراحي و اجرا گردید. روش‌ها: مطالعه توصیفی حاضر بر روی480 نفر از بیماران مبتلا به دیابت نوع 2 که بیماری آن‌ها توسط پزشکان متخصص شاغل در بیمارستان‌های تابعه دانشگاه علوم پزشکی تهران به تأیید رسیده بود با روش تصادفی در دسترس انجام گرفت. در این مطالعه از پرسش‌نامه‌ای دو قسمتی شامل اطلاعات وخصوصیات فردی بیماران و مرتبط با بیماری اخذ شد. جهت سنجش عادات تغذیه‌ای و ابعاد آن در دیابت در این بیماران از پرسش‌نامه استاندارد استفاده شد که با استفاده از 51 سؤال، 4 بعد مختلف عادات تغذیه‌ای در دیابت را در بیماران مبتلا اندازه‌گیری می‌نمود. این ابزار شامل 4 سؤال کلی در مورد عادات تغذیه‌ای، 12 سؤال مقیاس اطلاعات عمومی در دیابت، 6 سؤال در مورد برنامه‌ریزی، خرید و آماده کردن غذا، 17 سؤال در مورد عادات صرف غذا و 12 سؤال در مورد حمایت خانواده در عادات تغذیه‌ای در دیابت بود. جمع‌آوری اطلاعات به دست آمده اين مطالعه در یک مقطع زمانی و در چهار بیمارستان تابعه دانشگاه در سال 1391 انجام شد. برای تجزیه و تحلیل اطلاعات از نرم‌افزار SPSS نسخه 5/11 استفاده گردید و سطح معنی‌داری 05/0 در نظر گرفته شد. يافته‌ها: میانگین سنی شرکت کنندگان در مطالعه 53/11 ± 96/59 سال بود. بیشترین و کمترین میانگین امتیاز ابعاد چهارگانه ابزار سنجش عادات تغذیه‌ای در دیابت به ترتیب مربوط به ابعاد تأثیر خانواده بر عادات تغذیه‌ای (3/14 ± 72/64) و عادات خوردن غذا (13/12 ± 27/52) بود. در ابعاد مختلف این پرسش‌نامه مواردی از جمله توجه به برچسب مواد غذایی از نظر نوع و میزان کالری، توجه به صرف منظم وعده‌های اصلی و میان وعده‌های غذایی و دریافت حمایت از سوی خانواده بیشترین میانگین و انحراف معیار را کسب نمود. نتيجه‌گيري: با توجه به نتایج مطالعه پیشنهاد می‌گردد، به منظور ارتقای دانش و اتخاذ رفتارهای تغذیه‌ای بیماران مبتلا به دیابت نوع 2، مداخلات تئوری محور با هدف اصلاح عادات خوردن غذا طراحی و اجرا گردد. به نظر می‌رسد جلب مشارکت خانواده‌های بیماران مبتلا به دیابت به عنوان عوامل قادر کننده بتواند دستیابی به این هدف را تسهیل نماید.

Title: رابطه فرهنگ سازمانی و توانمندسازی کارکنان دانشگاه

http://hsr.mui.ac.ir/index.php/jhsr/article/view/885

مقدمه: بررسي فرهنگ سازماني مديران را قادر مي‌سازد تا با جمع‌آوري اطلاعات، بخش‌ها يا گروه‌هاي کاري را با يکديگر مقايسه، مسایل را الويت‌بندي و ادراکات و انتظارات کارکنان را شناسايي نمايند. همچنین شکاف بين وضع موجود و مطلوب را بهبود بخشند. هدف از نگارش مقاله حاضر بررسی رابطه فرهنگ سازمانی و توانمندسازی کارکنان ستادی دانشگاه علوم پزشکی اصفهان بود. روش‌ها: تحقیق حاضر از نوع توصیفی- پیمایشی بود. برای جمع‌آوری اطلاعات مربوط به ادبیات موضوع از روش كتابخانه‌اي نظيركتب و مجلات علمي و براي جمع‌آوري داده‌ها جهت تحليل و آزمون فرضيه‌هاي پژوهش از پرسش‌نامه محقق‌ساخته استفاده گرديد. روايي محتوایی آن توسط تعدادي از استادان و متخصصان تأييد شد. پايايي آن با استفاده از ضريب Cronbach’s alpha برابر 81 درصد و 90 درصد به ترتیب برای توانمندسازی و فرهنگ سازمانی محاسبه گردید. جامعه آماری این پژوهش 271 نفر از کارکنان ستادی دانشگاه علوم پزشکی اصفهان تشکیل می‌دادند که با استفاده از روش نمونه‌گیری تصادفی ساده بین 74 نفر پرسش‌نامه توزیع گردید. در این پژوهش برای تجزیه، تحلیل و آزمون فرضیه‌ها از نرم‌افزار SPSS استفاده شد. یافته‌ها: در نمونه مورد مطالعه، ابعاد اعتماد بین همکاران و استقلال با بعد رهبری سازمانی بیشترین همبستگی را داشت. همچنین رابطه معنی‌داری بین توانمندسازی با فرهنگ سازمانی وجود داشت و میزان این همبستگی 483/0+ بود. به عبارت دیگر با افزایش فرهنگ سازمانی میزان توانمندسازی شغلی نیز با ضریب 483/0 رو به افزایش می‌رود. نتیجه‌گیری: نتایج نشان داد که اگر فرهنگ سازمانی در راستای ارتقای توانمندسازی کارکنان باشد، افراد با انگیزه و اشتیاق بیشتری به انجام وظایف شغلی می‌پردازند. همچنین اگر از توانایی‌های کارکنان استفاده شود، امکان بروز خلاقیت و ابتکار عمل بیشتری وجود خواهد داشت.

Title: تأثیر استعمال سیگار بر افت شنوایی ناشی از مواجهه با صدا

http://hsr.mui.ac.ir/index.php/jhsr/article/view/847

مقدمه: اگر چه افت شنوایی ناشی از مواجهه با صدا مورد تأیید همه محققان است، اما ارتباط استعمال سیگار با افت شنوایی ناشی از صدا یکی از موضوعات قابل بحث می‌باشد. هدف از انجام این مطالعه، ارزیابی ارتباط استعمال سیگار با افت شنوایی ناشی از مواجهه با صدا بود. روش‌ها: در این مطالعه مقطعی که در سال 1389 در یک کارگاه صنعت لاستیک‌سازی صورت گرفت، 270 کارگر (135 نفر سیگاری و 135 نفر غیر سیگاری) مورد مطالعه قرار گرفتند. اندازه‌گیری صدا با دستگاه صداسنج 440CEL و ارزیابی افت شنوایی با استفاده از اتاقک آکوستیک و دستگاه ادیومتر 19Velton AD انجام شد. ارزیابی وضعیت استعمال سیگار و دیگر متغیرهای دموگرافیک با پرسش‌نامه‌ای که توسط محققین تکمیل می‌شد، انجام گردید. تحلیل داده‌های مطالعه با استفاده از نرم‌افزار SPSS نسخه16 انجام گرفت و 05/0 > P به عنوان سطح معنی‌داری در نظر گرفته شد. یافته‌ها: میانگین سن و سابقه شغلی افراد مورد مطالعه، به ترتیب 46/7 ± 16/34 و 89/6 ± 40/9 سال به دست آمد. در هر دو گروه در معرض صدای شغلی، میزان افت شنوایی در افراد سیگاری به طور معنی‌داری بیش از افراد غیر سیگاری بود. مدل رگرسیون نشان داد که افت شنوایی با متغیرهای سن، استعمال سیگار و مواجهه با صدا، ارتباط معنی‌دار خطی دارد. نتیجه‌گیری: اگر چه رابطه مصرف سیگار و افت شنوایی یک موضوع قابل بحث می‌باشد، اما یافته‌های این مطالعه نشان داد که مصرف سیگار در کنار مواجهه با صدا و عامل سن، به عنوان یک عامل خطر در تشدید افت شنوایی ناشی از صدا مطرح می‌باشد.

Title: شیوع خشونت‌های خانگی و عوامل مؤثر بر آن در زنان شهر اصفهان

http://hsr.mui.ac.ir/index.php/jhsr/article/view/1190

مقدمه: خشونت‌های خانگی رایج‌ترین شکل خشونت علیه زنان می‌باشد که تأثیر منفی بر سایر اولویت‌های بهداشتی مهم مانند سلامت مادران، تنظیم خانواده، پیشگیری از بیماری‌ها و بهداشت روان دارد و حتی سلامت کودکان را هم تحت تأثیر قرار می‌دهد. با در نظر داشتن این مهم که شیوع این سوء رفتارها در شهرهای مختلف کشور به لحاظ دارا بودن خصوصیات ویژه فرهنگی متفاوت می‌باشد؛ بنابراین مطالعه حاضر با هدف، تعیین شیوع خشونت‌های خانگی و عوامل مؤثر بر آن در زنان شهر اصفهان انجام شد. روش‌ها: پژوهش حاضر یک مطالعه توصیفی- تحلیلی بود که بر روی 900 نفر از زنان مراجعه کننده به مراکز بهداشتی- درمانی شهر اصفهان صورت گرفت. روش نمونه‌گیری در این مطالعه به صورت خوشه‌ای بود. جهت جمع‌آوری اطلاعات از پرسش‌نامه محقق ساخته مشتمل بر شش بخش (مشخصات فردی، سوء رفتار جسمی، سوء رفتار کلامی، سوء رفتار مالی، علل سوء رفتار و سوء رفتار روانی- عاطفی) و از رابطین مراکز بهداشتی- درمانی جهت جمع‌آوری اطلاعات استفاده شد. داده‌های جمع‌آوری شده توسط نرم‌افزار SPSS نسخه 11 و با استفاده از آمار توصیفی و استنباطی مورد تجزیه و تحلیل قرار گرفت و سطح معني‌داري 05/0 در نظر گرفته شد. یافته‌ها: میانگین سنی زنان مورد مطالعه 05/11 ± 1/34 سال و شیوع خشونت در زنان مورد مطالعه 44/34 درصد بود. در این مطالعه بین سن زنان و اعمال خشونت کلامی و مالی از جانب همسرانشان ارتباط آماری معنی‌داری دیده شد (05/0 > P). همچنین زنان با تعداد فرزند بیشتر، بیش از سایرین مورد خشونت مالی قرار گرفته بودند (001/0 > P). بین سن هنگام ازدواج با خشونت روانی- عاطفی و خشونت مالی ارتباط آماری معنی‌داری وجود داشت (01/0 > P). در این مطالعه زنان با سطح تحصیلات کمتر بیش از سایرین خشونت‌های کلامی و مالی را تجربه کرده بودند (001/0 > P). همچنین بین ابتلا به بیماری‌های روانی و انواع خشونت‌ها ارتباط آماری معنی‌داری دیده شد (01/0 > P). نتیجه‌گیری: آموزش و آگاه‌سازی زنان و مردان مهم‌ترین گام در پیشگیری و کاهش موارد خشونت علیه زنان است. ارایه برنامه‌های آموزشی مناسب با هدف ارتقای سطح آگاهی افراد جامعه؛ به طوری که بتوانند میان فرهنگ صحیح و بالنده ملی و مذهبی و سنت‌های غلط جاری زمینه‌ساز خشونت و صدمات جسمی و روحی تفاوت قایل شوند، ضروری به نظر می‌رسد.

Title: اجرای سیستم مدیریت کیفیت فراگیر (2000-9001 ISO) در ارتقای اثربخشی بیمارستان شهدای کرمانشاه

http://hsr.mui.ac.ir/index.php/jhsr/article/view/1337

مقدمه: پیاده‌سازی مدیریت کیفیت در بیمارستان می‌تواند منجر به ارتقای سطح عملکرد خدمت‌رسانی آن گردد. این مطالعه با هدف، بررسی تأثیر استقرار سیستم مدیریت کیفیت (2000-9001 ISO) بر اثربخشی خدمات در بیمارستان شهدای کرمانشاه انجام گرفت. روش‌ها: پژوهش حاضر یک مطالعه نیمه‌تجربی قبل و بعد بود که در بیمارستان شهدای کرمانشاه انجام گرفت. شاخص‌های متوسط زمان بستری، میزان عفونت بیمارستانی، مرگ و میر، رضایت بیماران و کارکنان قبل و بعد از مطالعه مورد سنجش قرار گرفت. داده‌ها توسط نرم‌افزار آماری SPSS نسخه 18 مورد تجزیه و تحلیل قرار گرفت. یافته‌ها: یافته‌ها حاکی از افزایش میانگین نمره رضایت کارکنان و بیماران و همچنین کاهش میزان عفونت‌های بیمارستانی، نسبت مرگ و میر به تعداد بیماران بستری شده و میزان متوسط مدت زمان بستری بعد از استقرار سیستم بود. نتیجه‌گیری: به نظر می‌رسد استقرار سیستم 2000-9001 ISO می‌تواند در ارتقای بهره‌وری بیمارستان مفید و از نظر اقتصادی یک طرح با فایده- هزینه مناسب باشد.
